# Blood Meal Analysis to Identify Reservoir Hosts for *Amblyomma americanum* Ticks

**DOI:** 10.3201/eid1603.090911

**Published:** 2010-03

**Authors:** Brian F. Allan, Lisa S. Goessling, Gregory A. Storch, Robert E. Thach

**Affiliations:** Washington University in St. Louis, St. Louis, Missouri, USA (B.F. Allan, L.S. Goessling, R.E. Thach); St. Louis Children’s Hospital, St. Louis (G.A. Storch)

**Keywords:** Amblyomma americanum, host blood meal identification, bacteria, white-tailed deer, ticks, reservoir capacity, vector-borne infections, zoonoses

## Abstract

Efforts to identify wildlife reservoirs for tick-borne pathogens are frequently limited by poor understanding of tick–host interactions and potentially transient infectivity of hosts under natural conditions. To identify reservoir hosts for lone star tick (*Amblyomma americanum*)–associated pathogens, we used a novel technology. In field-collected ticks, we used PCR to amplify a portion of the 18S rRNA gene in remnant blood meal DNA. Reverse line blot hybridization with host-specific probes was then used to subsequently detect and identify amplified DNA. Although several other taxa of wildlife hosts contribute to tick infection rates, our results confirm that the white-tailed deer (*Odocoileus virginianus*) is a reservoir host for several *A. americanum*–associated pathogens. Identification of host blood meal frequency and reservoir competence can help in determining human infection rates caused by these pathogens.

## Abstract 2

Blood meal analysis identified white-tailed deer as hosts for ticks that carry zoonotic pathogens.

Zoonotic pathogens, which reside in animal reservoir species and may at times spill over into human populations, are emerging at an unprecedented rate (*1*). Among these pathogens, several vector-borne pathogens have garnered considerable attention for the toll they exact on human health, which a growing body of evidence indicates may be exacerbated by anthropogenic environmental change (*2*–*4*). A rigorous understanding of the transmission dynamics of pathogens from infected wildlife hosts to vector organisms is critical to explorations of the ecology of vector-borne diseases.

Among the most rapidly emerging vector-borne zoonotic pathogens in the United States are several that are transmitted by the lone star tick (*Amblyomma americanum*). These pathogens include *Ehrlichia chaffeensis* and *E. ewingii*, both agents of human ehrlichiosis, and *Borrelia lonestari*, a potential agent of southern tick–associated rash illness (*5*). Ticks generally acquire pathogens by 2 primary modes of transmission: vertical (i.e., transovarial) transmission, whereby the pathogen is acquired maternally during development of the egg, and horizontally, whereby the pathogen is acquired through a blood meal on a reservoir-competent and infectious animal host. Recent research suggests that *E. chaffeensis* and *E. ewingii* are acquired horizontally (*6*,*7*); *B. lonestari* is likely transmitted horizontally and vertically (*8*). Several lines of evidence suggest that white-tailed deer (*Odocoileus virginianus*) are a major reservoir host for all 3 pathogens (*9*). Nonetheless, several other species have also been implicated as potential reservoirs, and our understanding of their relative roles in disease transmission remains incomplete.

Efforts to identify reservoir hosts for vector-borne zoonotic pathogens have historically been labor-intensive exercises, often requiring the capture of potential wildlife hosts, experimental infection with the pathogen of interest, and a subsequent examination of the efficiency with which these hosts pass the infectious agent to vector organisms under controlled conditions (*10*). However, such laboratory-based estimates may fail to capture the true distribution of host reservoir competencies because of unknown consequences of host selection behavior by vector organisms or the unmeasured contributions of cryptic reservoir hosts (*11*). An efficient solution has emerged in the form of host blood meal identification by molecular methods.

Because of the challenges posed by the duration of tick life cycles and host-seeking behavior, the feasibility of host blood meal identification in ticks was only recently established (*12*). Research efforts have converged upon a 2-step process: PCR amplification of and labeling with biotin any remnant vertebrate DNA isolated from a tick, and reverse line blot (RLB) hybridization whereby host-specific oligonucleotide probes are used to detect the biotin-labeled amplified host DNA. Several researchers have successfully used this technology to identify the reservoir hosts for numerous pathogens transmitted by *Ixodes ricinus*, a preeminent vector of tick-borne diseases in Europe (*13*–*16*). We describe the development of host-specific probes and the identification of host blood meals in wild-caught nymphal life stage *A. americanum* and present direct estimates of the reservoir capacity (an estimate of the absolute contribution of a reservoir host to the prevalence of infection in a tick population) for white-tailed deer and other reservoir hosts for the emerging *A. americanum*–associated zoonotic pathogens (*17*).

## Materials and Methods

### Field Collections

Questing *A. americanum* ticks were collected from 5 conservation areas and state parks in and surrounding St. Louis, Missouri, USA, during 2005 and 2007–2008. Ticks were collected either by dragging a 1-m^2^ white cloth along the ground and over vegetation or by using CO_2_-baited traps, whereby sublimating dry ice was used to attract ticks, which then became ensnared on double-sided carpet tape surrounding the trap. Both methods have proven effective for sampling nymphal and adult life stages of *A. americanum* (*18*). Captured ticks were removed and preserved in 70% ethanol for future identification and molecular analyses. Sampling efforts were limited to deciduous forested areas, which are the primary habitats in which *A. americanum* completes its life cycle (*5*). All subsequent analyses were limited to host-seeking nymphal life stage ticks, which for *A. americanum* are often presumed to have taken only 1 prior blood meal in the larval life stage.

### Laboratory Methods

#### DNA Extraction and Amplification

Nymphal life stage *A. americanum* were identified under a dissecting microscope before DNA extraction using the method of Kierans and Durden (*19*). Ticks were individually processed using 1 of 2 methods. All ticks collected in 2005 and most of those collected in 2007 were processed using the ammonium hydroxide method described previously by Pichon et al. (*13*). The remainder of the 2007 and all of the 2008 ticks were processed using a modified method described by Hammer et al. (*20*). The success of each method of DNA extraction was confirmed by PCR amplification and agarose gel electrophoresis of tick mitochondrial 16S rDNA as described (*21*,*22*).

Bacterial DNA was amplified in a multiplex PCR containing 2 sets of primers. Universal primers 0206 and 0209, previously described by Pichon et al. (*13*), were used to amplify a portion of the 16S rDNA, and primers 23SN2 and 5SCB, described previously by Rijpkema et al. (*23*), were used to amplify the 23S–5S intergenic spacer of the *Borrelia burgdorferi* complex. Primers 0209 and 5SCB were biotin labeled at the 5′ end to enable detection of the amplicons in the RLB assay. Primers were obtained from IDT (Coralville, IA, USA). Each set of amplification reactions contained at least 1 positive control (10 μL of known pathogen DNA) and 1 negative control (10 μL of DNA extraction negative control).

Vertebrate DNA was amplified by PCR using the biotin labeled primer 0049, described by Pichon et al. 2003 (*13*), and primer 0035 (5′-TTCTAGAGCTAATACATGCCRA-3′). These primers amplify a portion of the vertebrate (mammal and reptile) 18S rRNA gene. Primers were obtained from IDT. As with the bacterial DNA amplifications, at least 1 positive control (DNA from vertebrate tissue) and 1 negative control (negative DNA extraction control) were included with each set of PCRs.

#### Vertebrate Tissue DNA Extraction, Sequencing, and Probe Design

A small piece of vertebrate tissue, generally liver or muscle, was frozen on dry ice and then pulverized. The sample was then prepared using either the ammonium hydroxide or Chelex method. The resulting supernatant was removed to a fresh tube and a dilution of this supernatant was used in the PCRs.

Primers 0066 and 0067 (*13*) were used to amplify a 350–400-bp fragment of the vertebrate 18S rRNA gene. This fragment contains the area amplified by primers 0049 and 0035. Primers were obtained from IDT. PCR products were purified by using the Wizard SV Gel and PCR Clean-Up System (Promega Corporation, Madison, WI, USA). The purified amplicons were double-strand sequenced by using primers 0066 and 0067 by the Protein and Nucleic Acid Chemistry Laboratory at Washington University with ABI Prism Dye Terminator BigDye Premix version 1.1 (Applied Biosystems, Foster City, CA, USA).

MegAlign and EditSeq softwares (DNASTAR, Inc., Madison, WI, USA) were used to align and edit sequence data. The obtained sequences were aligned with 18S sequences found in GenBank and areas of variability were used to design probes.

#### Reverse Line Blot Hybridization

An RLB assay was used to identify bacterial DNA amplified from the tick lysates. In the assay, biotin-labeled PCR products are hybridized against a set of bacteria-specific probes (Table 1) that have been covalently linked to an activated Biodyne C membrane (Pall, Ann Arbor, MI, USA) by their 5′ amino group. Our method is based on RLB techniques previously described (*13*,*23*).

**Table 1:** Oligonucleotide sequences of bacterial probes used in reverse line blot assay

Probe ID	Nucleotide sequence (5′ → 3′)	Target organism (RNA genes)	Reference sequence
Ptg011	aacatgaacatctaaaaacataaa	*Borrelia garinii* (23S–5S)	*
Ptg012	AACATTTAAAAAATAAATTCAAGG	*B. afzelii* (23S–5S)	*
Ptg013	CATTAAAAAAATATAAAAAATAAATTTAAGG	*B. valaisiana* (23S–5S)	*
Ptg009	CTTTGACCATATTTTTATCTTCCA	*B. burgdorferi* s.l. (23S––5S)	*
Ptg010	AACACCAATATTTAAAAAACATAA	*B. burgdorferi* s.s. (23S–5S)	*
Ptg003	CGAACTTCTGGGTCAAGAC	*B. burgdorferi* s.l. (16S)	†
Ptg020	AGATAACTACTCTCCGTTTG	*B. lonestari* (16S)	AY166715
Ptg022	TCCTAATAGGGGGAGTC	*Ehrlichia chaffeensis* (16S)	M73222
Ptg023	CTTTTAACAGAGGGAGTCA	*E. ewingii* (16S)	M73227
Ptg024	TCCTAACAGGGGGAGTC	*E. canis/ovina/muris* (16S)	AY394465, AY318946, ABO13009
Ptg007	TGGGGATTTTTTATCTCTGTG	*Anaplasma phagocytophilum* (16S)	†
Ptg021	CTACCACTGACGCTGAT	*Rickettsia rickettsii* (16S)	DQ150694
Ptg027	CTTCGGAACGCAGTGAC	*Francisella tularensis* *+ F. philomiragia* (16S)	Z21932, Z21933
Ptg026	CTTGGGGAGGACGTTAC	*F. tularensis* subsp. *tularensis* (16S)	Z21932
Ptg029	GCCTATRAGTTAATAGCTTGT	*F. philomiragia* (16S)	Z21933
Ptg028	TCCTGCGATCTTTCTAGA	*F. endosymbiont* of Dv (16S)	AF166256
Ptg032	CATCCAGGGAAGTAAGC	*Arsenophonus* spp. (16S)	AY265347
Ptg030	GCTACAACTGACACTGATG	*R. endosymbiont* of Dv (16S)	AY375427
Ptg031	TACAACTGACGCTAATGC	*R. amblyommii* + *Rickettsia* sp. (16S)	U11012
Ptg035	TCGGAAGATTATCTTTCGG	*R. amblyommii* (16S)	U11012

*Designed by Rijpkema et al. 1995 (*23*). †Designed by Pichon et al. 2003 (*13*).

The probes were applied in lines to an activated membrane using a Miniblotter 45 (Immunetics, Cambridge, MA, USA). The membrane was stored at 4°C until use. Before starting the hybridization, the membrane was incubated in hybridization buffer (0.3 mol/L sodium chloride, 0.02 mol/L sodium phosphate buffer, 0.002 mol/L EDTA, 0.1% sodium dodecyl sulfate) for 45 min at 42°C. For the hybridization step, the membrane was placed in the Miniblotter with the orientation shifted 90° so that the probe lanes were aligned perpendicular to the slots. Each slot was filled with 140 μL of denatured biotinylated PCR products (10 μL PCR products in 140 μL hybridization solution, heated at 99°C for 10 min, then cooled on ice) and incubated at 42°C for 90 min. The PCR solutions were aspirated off and the membrane was washed twice with hybridization buffer at room temperature, then twice at 50°C with preheated buffer. Biotin-labeled PCR products hybridized to probes were detected using the CDP-Star Universal Detection Kit (Sigma, St. Louis, MO, USA) and exposure to Blue Ultra Autorad film (ISC BioExpress, Kaysville, UT, USA).

A second RLB assay using host specific probes was used to identify vertebrate DNA amplified from the tick lysates (Table 2). The protocol for the vertebrate RLB was the same as for the bacterial RLB except the prehybridization wash, hybridization and high stringency wash steps were all conducted at 62°C.

**Table 2:** Oligonucleotide sequences of vertebrate probes used in reverse line blot assay

Probe ID	Probe name	Nucleotide sequence (5′ → 3′)	Reference sequence
PRNA010	Aves	ccgacctccggggacgc	*
PRNA012	Passeriformes	GGGCCCGCCCGGCAGCT	*
PRNA029	Galliformes	GGGCTCGCCCGGCGGCT	*
PRNA042	Squamata/testudines	CGCTGACCTCCGGGGATGC	*Sceloporus undulatus* (M36359, M59400), *Crotaphytus collaris collaris*† (FJ797666)*, Trachemys scripta*† (FJ797668)*, Scincella lateralis* (AY217908), *Eumeces fasciatus* (AY217920), *Elaphe obsoleta*† (FJ797667), *Heterodon platirhinos* (M59392)
PRNA043	Amphibia	CGCTGACCCCCAGGGATGC	*Rana amurensis* (AF542043), *R. chensinensis* (AY145522), *Xenopus laevis* (X04025)
PRNA045	Ruminantia	GGTCAGCCTCCTCCCGGC	*Odocoileus* virginianus† (FJ797665)*, Capreolus capreolus* L (AY150545), *Cervus elaphus* L (AY150547), *Bos taurus* (AY779625)
PRNA018	Leporidae	CGGGGGGGTGGGCGCCG	*
PRNA047	Leporidae/carnivore	GGTCAGCCTCCCCCCGGC	*Sylvilagus floridanus* (FJ797663)*, Procyon* lotor† (FJ797659), *Felis catus* L (AY150542),
PRNA046	Canidae	GGTCAGCCTCCCTCCGGC	*Canis latrans*† (FJ797662), *Canis lupus familiaris*† (FJ797658), *C. lupus familiaris* (DQ287955), *Vulpes vulpes* (AY150549)
PRNA026	Sciurus	CGGTCAGCTTCCCCCCGG	*
PRNA037	Blarina	AGCCTCCCCTCGGCTCCG	*Blarina* sp.† (FJ797661)
PRNA030	Erinaceus	CTCCCTCCGGCTCCGGC	*
PRNA017	Myodes 1	GAGCTCCCCCGCGGCCC	*
PRNA050	Myodes 2	CGACGGGCGCCGACCCC	*Myodes glareolus* (AY150543)
PRNA011	Murinae/gerbilinae	CCCTCCCGGCTCCGGCCG	*
PRNA034	Rattus	CGGTCAGCCCCCTCCCGG	*Rattus norvegicus* (X01117)
PRNA033	Mus	CCGGTGAGCTCCCTCCCGG	*Mus musculus* (X00686)
PRNA035	Sigmodontinae	TCAGCTCCCTCCCGGCCCC	*Peromyscus* sp.† (FJ797660), *Peromyscus leucopus* (AY591913)
PRNA032	Didelphis	CGGCGGCTTCCCCCTAACC	*Didelphis virginiana* (J311677)
PRNA048	Mephitis	GGTCAGCCTCTCCCCGGC	*Mephitis mephitis*† (FJ797664)

*Designed by Pichon et al. 2003 (*13*). †Sequence obtained in this study.

#### Tick Identification

To confirm correct identification of *A. americanum* nymphs used in our study, we selected 4 tick samples for which we amplified and then double-strand sequenced a portion of the tick 16S rRNA gene. The 16S+1 and 16S-2 primers described in Black and Piesman (*21*) were used for PCR amplification and sequencing.

#### Statistical Analyses

All statistics were calculated using Poptools version 3.0 in Microsoft Excel (Microsoft, Redmond, WA, USA) (*24*). We used χ^2^ tests with the Yates continuity correction to analyze patterns of pathogen co-infection and the distributions of blood meals among hosts. We estimated 95% confidence intervals for our estimates of reservoir capacity based upon identifiable blood meals using the Wilson score method without continuity correction.

## Results

### Pathogen Detection

Three of the most widely reported pathogens associated with *A. americanum* (*E. chaffeensis*, *E. ewingii,* and *B. lonestari*) were detected among collections from >3 of 5 study sites (i.e., each pathogen was detected from ticks collected at >3 locations). Of the 1,383 nymphal life stage *A. americanum* ticks tested, 19 (1.4%) contained *E. chaffeensis*, 31 (2.2%) contained *E. ewingii*, and 18 (1.3%) contained *B. lonestari*. No co-infections with >1 pathogen were detected in any tick. However, χ^2^ analyses for each pair of pathogens indicated that this outcome did not differ from random chance (*E. chaffeensis* and *E. ewingii*: χ^2^ = 0.013, df = 1, p = 0.908; *E. ewingii* and *B. lonestari*: χ^2^ = 0.024, df = 1, p = 0.877; *B. lonestari* and *E. chaffeensis*: χ^2^ = 0.359, df = 1, p = 0.549).

#### Host Probes

DNA from 13 vertebrate species (for which sequences of 18S rDNA were not available in the GenBank database) were purified and subsequently amplified for sequencing. The amplicons were double-strand sequenced and these sequences together with those available in the GenBank database were aligned to generate vertebrate host probes (Table 2). Eventually, 20 host probes were established, and 34 vertebrate species that were identified from the literature as potentially important hosts were correctly identified to the matching host probe, with 1 exception (*Tamias striatus* reacted with Canidae probe) (Table 3).

**Table 3:** Hybridization by host DNA to vertebrate reverse line blot probes

Probe ID	Probe name	Vertebrate DNA hybridized
PRNA010	Aves	*Turdus migratorius, Meleagris gallopavo, Gallus gallus, Chen caerulescens*
PRNA012	Passeriformes	*T. migratorius*
PRNA029	Galliformes	*M. gallopavo, G. gallus, C. caerulescens*
PRNA042	Squamata/testudines	*Crotophytus collaris, Elaphe obsoleta, Trachemys scripta elegans*
PRNA043	Amphibia	*Rana clamitans*
PRNA045	Ruminantia	*Odocoileus virginianus, Cervus elephus, Bos taurus, Sus scrofa domestica*
PRNA018	Leporidae	*Sylvilagus floridanus, Sus scrofa domestica*
PRNA047	Leporidae/carnivora	*S. floridanus, Felis catus, Procyon lotor*
PRNA046	Canidae	Canis lupus familiaris, C. latrans, Vulpes vulpes, Tamias striatus*
PRNA026	Sciurus	*Sciurus carolinensis, Sciurus niger, S. griseus, Marmota monax*
PRNA037	Blarina	*Blarina brevicauda, Sorex vagrans*
PRNA030	Erinaceus	No hybridization with any vertebrate DNA tested
PRNA017	Myodes 1	*Myodes gapperi*
PRNA050	Myodes 2	*M. gapperi, Microtus californicus*
PRNA011	Murinae/gerbilinae	*Rattus norvegicus, Mus musculus, Zapus hudsonius*
PRNA034	Rattus	*Rattus norvegicus*
PRNA033	Mus	*M. musculus*
PRNA035	Sigmodontinae	*Peromyscus* spp., *Neotoma fuscipes*
PRNA032	Didelphis	*Didelphis virginiana*
PRNA048	Mephitis	*Mephitis mephitis*

*The reaction was confirmed by using 2 tissue samples. The PCR amplicon was sequenced and matches the Canidae probe.

### Detection of Host DNA

Purified lysates from all 1,383 nymphal life stage *A. americanum* screened for pathogenic microbes in the previous analyses were also subjected to host blood meal identification. Remnant host DNA from 869 (62.8%) of these ticks hybridized with 10 of the 20 host probes used (Table 4). Of these samples, 389 (44.8%) hybridized to the Ruminantia probe, which for wildlife hosts in the St. Louis, Missouri, region is likely limited to white-tailed deer (Table 3). The remaining blood meals were distributed across a variety of taxa. DNA from more than 1 host was detected in 141 nymphal life-stage ticks (Table 4).

**Table 4:** Identification of host DNA in questing *Amblyomma americanum* nymphs, Missouri, USA, 2005 and 2007–2008

Host data	2005	2007	2008	All
No. nymphs analyzed (no. hosts identified)	75 (33)	489 (240)	819 (596)	1,383 (869)
No (%) nymphs				
Ruminantia	5 (15.2)	147 (61.3)	237 (39.8)	389 (44.8)
Galliformes	4 (12.1)	16 (6.7)	77 (12.9)	97 (11.2)
Passeriformes	1 (3.0)	17 (7.1)	76 (12.8)	94 (10.8)
Sciurus	17 (51.5)	13 (5.4)	65 (10.9)	95 (10.9)
Leporidae	3 (9.1)	3 (1.3)	15 (2.5)	21 (2.4)
Squamata/testudines	0	15 (6.3)	3 (0.5)	18 (2.1)
Canidae	0	1 (0.4)	7 (1.1)	8 (0.9)
Leporidae/carnivora	0	0	3 (0.5)	3 (0.3)
Sigmodontinae	1 (3.0)	1 (0.4)	1 (0.2)	3 (0.3)
Mixed	2 (6.1)	27 (11.3)	112 (18.8)	141 (16.2)

Of the 68 *A. americanum* nymphs containing pathogenic bacteria, 47 (69.1%) contained identifiable vertebrate DNA (i.e., that hybridized with >1 host probe; Table 5). Of the 15 *E. chaffeensis*–positive samples that contained identifiable vertebrate DNA, 8 hybridized only with the Ruminantia probe, and 4 others hybridized with the Ruminantia probe plus ≥1 additional probes; thus 12 of 15 identifiable samples hybridized with the Ruminantia probe. The other identifiable *E. chaffeensis*–positive samples hybridized either with the Sciurus (n = 2) or the Leporidae (n = 1) probes. For the 23 identifiable *E. ewingii*–positive samples, 12 contained DNA that hybridized only with the Ruminantia probe, 3 that hybridized only with the Sciurus probe, and 1 that hybridized only with the Leporidae probe. All 6 of the identifiable mixed blood meal DNAs hybridized with ≥2 of these 3 host probes. The remaining identifiable *E. ewingii*–positive sample hybridized only with the Passeriformes probe. For the 9 identifiable *B. lonestari*–positive samples, 4 hybridized with the Ruminantia probe, 1 hybridized with the Sciurus probe, 1 hybridized with the Passeriformes probe, and 1 hybridized with the Squamata/Testudines probe (which is expected to detect DNA from lizards, snakes, and turtles).

**Table 5:** Blood meal source in pathogen-positive *Amblyomma americanum* nymphs, Missouri, USA, 2005 and 2007–2008

Host	No. *A. americanum* nymphs infected		
	*Ehrlichia chaffeensis*	*E. ewingii*	*Borrelia lonestari*
Ruminantia	8	12	4
Sciurus	2	3	1
Leporidae	1	1	0
Passeriformes	0	1	1
Squamata/testudines	0	0	1
Mixed	4	6	2
Not identified	4	8	9
Total	19	31	18

Because there is evidence that *B. lonestari* can be transovarially transmitted (*8*), it is crucial to test whether the associations between host blood meals and pathogen infections differ from a distribution expected by random chance alone. The frequency of association between *B. lonestari* infection and the Ruminantia probe (χ^2^ = 0.033, df = 1, p = 0.855), the Sciurus probe (χ^2^ = 0.217, df = 1, p = 0.641), the Passeriformes probe (χ^2^ = 0.209, df = 1, p = 0.647), and the Squamata/Testudines probe (χ^2^ = 0.639, df = 1, p = 0.424) did not differ from a distribution expected by random chance. Owing to the detection of host blood meals in pathogen-positive and pathogen-negative ticks, we were able to generate estimates of reservoir capacity (calculated as the proportion of blood meals from a given host that result in an infection for a given pathogen and includes the end products of tick feeding and molting success) for each taxonomic grouping of reservoir host and pathogen species (Table 6).

**Table 6:** Estimates of reservoir capacity for reservoir hosts of *Amblyomma americanum*–associated zoonoses*

Host	% Blood meals associated with pathogen infection (95% CI)	
	*Ehrlichia chaffeensis*	*E. ewingii*
Ruminantia	2.1 (30 –75.2)	3.1 (33.0–70.8)
Sciurus	2.1 (3.7–37.9)	3.2 (4.5–32.1)
Leporidae	4.8 (1.2–29.8)	4.8 (0.8–21.0)
Passeriformes	0	1.1 (0.8–21.0)
Squamata/testudines	0	0

*CI, confidence interval. *Borrelia lonestari* is omitted because of the confounding influence of transovarial transmission.

### Tick Identification

Two of the tick samples analyzed contained DNA that reacted with the Squamata/Testudines probe, 1 of which was also positive for *B. lonestari*, and 2 samples contained DNA that reacted with the Passeriformes probe, 1 of which was also positive for *E. ewingii*. The sequences obtained from the 4 ticks were identical except for an extra basepair in 2 of the sequences. The sequences were compared with 16S sequences of other potential tick species in Genbank and had 98%–100% homology with *A. americanum* sequences, but only 84% homology with *Haemaphysalis* *leporispalustris* and 81% homology with *A. tuberculatum*.

## Discussion

Three of the zoonotic pathogens primarily associated with *A. americanum* (*E. chaffeensis*, *E. ewingii*, and *B. lonestari*) were detected at our field sites at infection rates in nymphal life stage ticks comparable to levels reported elsewhere in the region (*25*,*26*). Our array of host probes indicates that *A. americanum* feed from a variety of vertebrate hosts in the larval life stage, consistent with observations from field studies (*5*). We found that most nymphal *A. americanum* infected with *E. chaffeensis* fed upon a white-tailed deer in the larval stage, consistent with the prevailing hypothesis that this is the major wildlife reservoir for this emerging pathogen (*9*). Analysis of 3 other *E. chaffeensis*–positive blood meals associated with the Sciurus and Leporidae probes suggests that members of the genus *Sciurus* (likely fox and gray squirrels, *S. niger* and *S. carolinensis*, respectively) and eastern cottontail rabbits (*Sylvilagus floridanus*) may also function as wildlife reservoirs for *E. chaffeensis*. Most blood meals detected from *E. ewingii*–positive ticks were also associated with the Ruminantia, Sciurus, or Leporidae probes. Considering the lack of evidence for transovarial transmission of *E. chaffeensis* (*6*) and *E. ewingii* (*7*), we consider the wildlife hosts in these taxa to be the major reservoir hosts in this region.

No consistent associations between the sources of host blood meals and infection rates with *B. lonestari* in nymphal life stage ticks were found. In light of evidence that *B. lonestari* can be transovarially transmitted (*27*), it may not be possible to determine whether an infected tick acquired this pathogen through a blood meal from an infective host or through vertical transmission from mother to offspring. Therefore, host blood meal identification may not be an adequate means to identify reservoir hosts for this pathogen. Increased samples sizes combined with knowledge of transovarial transmission rates may eventually enable researchers to quantify the contributions of reservoir hosts to infection prevalence of *B. lonestari* in *A. americanum*.

Our data enable us to further generate estimates of reservoir capacity, defined as the absolute contribution of a reservoir host to the prevalence of pathogen infection in a tick population. This metric includes the influence of host abundance, the probability that a host is infected, infectivity of that host, and tick feeding and molting success rates (*17*). Although this metric should not be mistaken for an estimate of actual reservoir competence (i.e., the proportion of ticks that become infected from feeding on infective hosts), it may be more informative because it includes the outcome of several ecologic processes that ultimately determine human risk of exposure to tick-borne pathogens. We found that white-tailed deer do not yield the highest absolute estimates of reservoir capacity for any of the 3 pathogens in our study. However, estimated confidence intervals suggest this outcome may be due to small sample sizes for estimates of reservoir capacity for other reservoir hosts. In light of evidence that white-tailed deer are often infected with these pathogens throughout the range of *A. americanum* ticks (*28*–*30*), we hypothesize that white-tailed deer may be weakly competent reservoirs for these pathogens. However, when taking into account the frequency with which *A. americanum* encounter these abundant hosts, (i.e., reservoir potential) (*30*), it remains apparent that white-tailed deer are major reservoir hosts for *A. americanum*–associated zoonoses.

From the nymphal life stage *A. americanum* that yielded detectable host DNA in this study, 16.2% hybridized with >1 taxonomic probe. Mixed blood meals, presumably caused by bouts of interrupted feeding, have been reported from other studies on ixodid ticks using host blood meal identification, at similar rates to those reported here (*15*,*16*). For example, Morán Cadenas et al. reported multiple host detections from 19.2% of detectable blood meals in *Ixodes ricinus*, with no differences between nymphal and adult life stages (*15*). The absence of a detectable blood meal in 37.2% of the *A. americanum* nymphs examined in our study is also consistent with results from other studies using host blood meal identification in ixodid ticks (*13*–*16*). We speculate that the degradation of remnant host DNA is the primary cause of this phenomenon, because our ability to detect host blood meals declined later in the season (unpub. data).

It is crucial to temper our conclusions about the role of various hosts derived from our data with some exploration of other factors that may influence the outcome of host blood meal identification. Various factors may influence the detectability of host blood meals, such as the presence of nucleated erythrocytes, host blood volume, permissiveness of hosts (a measure of the ability of a tick to successfully feed to repletion on a given host), and the region of DNA targeted for analysis (*12*). Because the first step of the PCR in our study is subject to dominant template bias, remnant DNA from nucleated erythrocytes may mask mammalian DNA present in mixed blood meals. Additionally, we did not directly quantify the sensitivity of our various host probes, although we did attempt to identify host probe concentrations that yielded equivalent reactions. Nonetheless, variation in host probe sensitivity may introduce another source of error in our findings. In light of these potential limitations to host blood meal identification, field-based studies will remain necessary in order to determine if host blood meal distributions are consistent with the availability of hosts and host-vector interactions.

Host blood meal identification by molecular methods offers a direct and efficient approach for understanding the contributions of both reservoir competent and incompetent hosts to the transmission dynamics of tick-borne diseases. Through this emerging technology, we show the major role played by white-tailed deer in facilitating the emergence of *A. americanum*–associated zoonoses. However, the apparent contributions of various other hosts to pathogen transmission highlight the need for a community approach to understanding vector-borne zoonoses. Future applications of these methods will generate information for approaching a variety of topics of pressing concern to public health, including the potential impact of anthropogenic landscape change on human risk of exposure to zoonotic pathogens.
